# Effect of the Concentration of SrAl_2_O_4_: Eu^2+^and Dy^3+^ (SAO) on Characteristics and Properties of Environment-Friendly Long-Persistent Luminescence Composites from Polylactic Acid and SAO

**DOI:** 10.1155/2021/6337768

**Published:** 2021-09-27

**Authors:** Zhongjin Ni, Tianyu Fan, Shuyang Bai, Shiyu Zhou, Yan Lv, Yihua Ni, Bin Xu

**Affiliations:** ^1^College of Engineering, Zhejiang A&F University, Linan 311300, China; ^2^College of Materials Science and Engineering, Zhejiang University of Technology, Hangzhou 310014, China

## Abstract

We report luminous polylactic acid (PLA) composite prepared via a solvent casting method using different amounts of phosphor strontium aluminate (SrAl_2_O_4_: Eu^2+^ and Dy^3+^) (SAO). The reason for doing this is that the changes of fluorescence and mechanical properties in the composites with different SAO contents can be directly evaluated. The SAO particles should have a variety of excellent characteristics in the PLA matrix, among which dispersibility and compatibility are particularly important; so, they can be modified by 3-aminopropyltriethoxysilane (APS) to achieve the target characteristics. The results showed that the fluorescence and mechanical properties were affected by SAO addition. The mechanical properties significantly improved with 5 wt% SAO; further, addition had no impact. And the emission band of fluorescence and phosphorescence is just at the peak of 524 nm. The composites with 15 wt% SAO have the best fluorescence properties. The fluorescence decreased with further doping. Fluorescence decay curves with various amounts of SAO particles show a similar tendency as pure SAO particles; the speed of decrease in afterglow intensity was higher for the first 30 min. In addition, the detailed morphological scanning and study by scanning electron microscope (SEM) showed that the particles had good adhesion to the matrix. In conclusion, the concentration of SAO into the PLA matrix impacts the fluorescence and mechanical properties of a SAO/PLA composite material.

## 1. Introduction

Long persisting luminescent (LPL) materials have an important feature, and they can continue to emit light for a long time (even hours) after excitation; so, they are often used as inorganic photoluminescent materials, and they are also widely used in energy storage materials and because of the specificity of this characteristic, LPL materials—especially aluminates like SrAl_2_O_4_: Eu^2+^ and Dy^3+^ (SAO) widely used in luminescent ceramics and coatings, as well as in emergency escape clothing and signs [1]. Because of their high luminous efficiency, long persistence time, stable chemical properties, and lack of radiation pollution, SAO phosphors are widely developed [2]. Biodegradable polymers like polylactic acid (PLA) are frequently used with SAO [3, 4]. This sustainable and environment-friendly material has a good prospect in medical materials, home decoration materials, packaging materials, and other fields [5]. Although PLA has excellent processability in many equipment and offers good attachment and strength, however, it is frangible and brittle, which limits its use. Thus, PLA should be modified for most practical applications [4, 6].

Luminous PLA composites can be prepared with SAO to improve their mechanical properties. These composites have excellent phosphorescence, fluorescence, plasticity, elasticity, and self-degradation [7]. Over the last few years, this material has gained a lot of interest in areas such as radiation detection [8], industrial applications [9, 10], and in vivo imaging [11]. This preserves the properties of the aluminates and improves PLA's mechanical properties.

Here, SAO particles were added to a PLA composite. Samples were processed via solvent casting using different amounts of SAO particles. The effects of SAO content on the microstructure, thermal, tensile, and afterglow properties of SAO/PLA composite were investigated in detail. To enhance the behavior of these composites, SAO particles were treated with a 3-aminopropyltriethoxysilane (APS) coupling agent [12]. The treated SAO particles were then doped at 5, 10, 15, and 20 wt% in a copolymer solution (polyethylene glycol was added to increase the compatibilization of SAO in the PLA matrix). The final product was prepared via solvent casting with characterization of optical properties, mechanical properties, and morphological characteristics.

## 2. Materials and Methods

### 2.1. Materials

PLA 4032D was purchased from Nature Works Company. Long-persistent luminescent materials doped with strontium aluminate (SrAl_2_O_4_:Eu^2+^ and Dy^3+^; SAO) were provided by Zhejiang Minghui Luminescence Technology Company. Polyethylene glycol 2000/PEG2000 was provided by Shanghai Yuanye Biotechnology Company. Dichloromethane solution was provided by Shanghai Aladdin Biotechnology Company. Anhydrous ethylene alcohol was supplied by China Pharmaceutical Group Chemical Reagent Company. Silane coupling agent APS (KH550) was purchased from Linan Karl Biotechnology Company.

### 2.2. SAO/PLA Composite Material Preparation and Methods

#### 2.2.1. Modification of SAO Particles

**Scheme 1:**
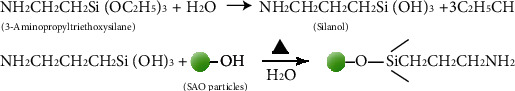
Hypothetical reaction of KH550 and SAO particles.

**Scheme 2:**
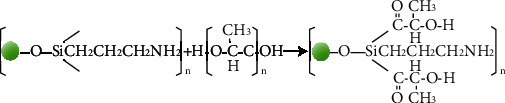
Crosslinking reaction of silylated SAO with PLA.

The deionized water (20 ml), absolute ethanol (180 ml), and KH550 (2 wt% of the total weight) were added to a 400 ml beaker and mixed by a magnetic stirrer at a speed of 500-700 r/min for 60 min. The sample was magnetic stirred for 1 hour at a speed of 500-700 r/min. The above solution added with SAO was stirred under 60°C at 500-700 r/min. Ethoxy is a special group that can be hydrolyzed in water or solvent to form silanol, and it is also a group contained in KH550. The -OH group of PLA can react with silanol, which in turn makes a stable covalent bond formed on the surface of the PLA polymer [13, 14] (Schemes 1 and 2). The treated SAO was vacuum filtered in a drying box at 80°C for 6 hours.

#### 2.2.2. Preparation of SAO/PLA Composite Materials

The PLA powder was dried for 24 hours at a temperature of about 70 degrees Celsius, and then the solution was stirred adding 0.1 g/mol PLA powder into dichloromethane for 1 hour at 1000-2000 r/min followed by 0.5 g polyethylene glycol and pretreated SAO powder into the solution. Polyethylene glycol disperses the SAO particles to facilitate attachment to the PLA surface [15]. Next, copolymer solutions containing 5, 10, 15, and 20 wt% SAO particles were melt spun. These solutions were stirred by ultrasonic stirring for 30 min, and then each group of solution samples was kept for 12 hours and dried at 60°C for 24 hours.

### 2.3. Testing and Characterization

#### 2.3.1. XRD Analysis

The XRD patterns of the polymers were collected on a SHIMADZU XRD-6000 diffractometer with a scanning speed of 2°/min (2*θ* from 5° to 60°) using a nickel-filtered Cu-*Κα* radiation (*λ* = 1.54056 Å) as the radiation source (40 kV, 40 mA).

#### 2.3.2. FT-IR Analysis

At room temperature, the infrared spectrum of each powder sample sandwiched between two KBr particles was recorded through a Perkin-Elmer 1100 Model with a wavelength range of 4000 cm^−1^ to 500 cm^−1^ (the spectrum was measured using 32 scans at 4 cm^−1^ resolution).

#### 2.3.3. DSC Analysis

The Netzsch STA 409PC Maia instrument was used for DSC analysis in a nitrogen atmosphere (purge flow rate of 50 mL/min). In order to obtain the curing heat flow curves of the powder samples, different heating rates (such as 5, 10, 15 and 20 K/min) were used in the measurement, while dynamic DSC measurements were made in the thermal sweep range from 40°C to 250°C. Each new sample is about 5-8 mg in an aluminum tray protected by N_2_ gas.

#### 2.3.4. TGA Analysis

A thermogravimetric analysis was performed on each sample with an initial weight of approximately 10 mg using the STA 409PC by storing the samples in an open Pt pan with high purity nitrogen as purge gas and raising the temperature from room temperature to 800°C at a heating rate of about 20°C/min.

#### 2.3.5. Tensile Property Test

An electronic universal testing machine (with a 2 KN load cell) manufactured by Meister Industrial Systems Company was used to test the tensile properties of the sample at a crosshead speed of 2 mm/min. Each sample measured 75 mm × 4 mm × 1 mm. A total of 10 samples were used in the experiment and tested repeatedly.

#### 2.3.6. SEM Analysis

Under the acceleration voltage of 15000 V, through Hitachi SU8010 (S-4800-1) field emission scanning electron microscope and then scanning SEM micrographs imaging of the fracture morphology of the impact specimens, the gold thickness was 10 nm. The fracture surfaces were coated with a thin evaporated layer of gold.

#### 2.3.7. Fluorescence Spectrum Analysis

The sample was irradiated for 12 hours under ultraviolet light (wavelength range 320-400 nm, central wavelength 360 nm, power 60 *μ*W/cm^2^). A fluorescence spectrophotometer F97PRO (Shanghai Prism Light Technology Company) was used to measure the fluorescence spectra. The peak value is 524 nm (*λ* = 524 nm). The decay curves were obtained by measuring continuously for 1 hour. The fluorescence emission spectra at room temperature and phosphorescence spectra at 1 min were obtained by excitation of the sample with a wavelength of 360 nm.

## 3. Results and Discussion

### 3.1. Crystallization Behavior

**Figure 1:**
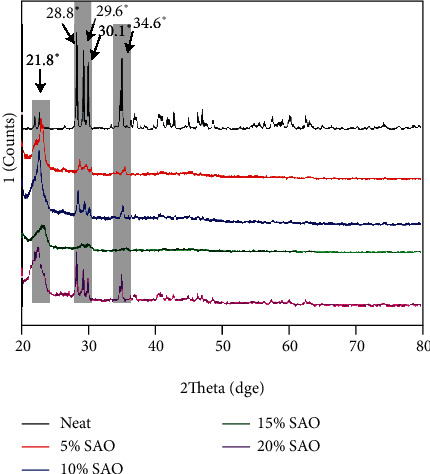
XRD patterns of pure PLA, 5 wt% SAO/PLA, 10 wt% SAO/PLA, 15 wt% SAO/PLA, and 20 wt% SAO/PLA.

Figure 1 shows XRD patterns of neat PLA, 5 wt% SAO/PLA, 10 wt% SAO/PLA, 15 wt% SAO/PLA, and 20 wt% SAO/PLA composites. The peaks at 2*θ* = 21.8°, 28.8°, 29.6°, 30.1°, and 34.6° are present in XRD patterns of SAO particles indicating that the crystal types of the SAO/PLA composite changed upon addition of SAO particles, and its characteristic peak obviously decreased. Moreover, the characteristic peak of PLA/SAO composite containing 15 wt% SAO almost disappeared, which was related to the strontium aluminate phosphor particles [16]. This means that the addition of SAO particles enhances the elongation at break and decreases the melting point, barrier property, and heat resistance.

### 3.2. FT-IR Spectra

**Figure 2:**
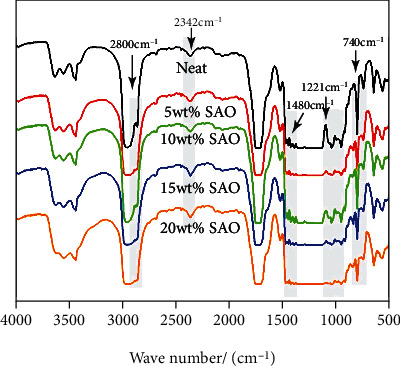
FT-IR spectra of pure PLA, 5 wt% SAO/PLA, 10 wt% SAO/PLA, 15 wt% SAO/PLA, and 20 wt% SAO/PLA.

The FT-IR spectra of pure PLA, 5 wt% SAO/PLA, 10 wt% SAO/PLA, 15 wt% SAO/PLA, and 20 wt% SAO/PLA composites are shown in Figure 2. Two distinct peaks at 2800 cm^−1^ and 1221 cm^−1^ demonstrating the presence of -OH groups indicating that the addition of SAO particles enhances crosslinking between SAO and PLA. The specific FT-IR spectrum diagram is shown in Figure 2. Peaks at 2342 cm^−2^ proved that the SAO is properly copolymerized on PLA. The 1450-1480 cm^−1^ band is weakening vibration of the -CH_3_ group, which shows SAO particles transforming into the luminous SAO/PLA particles. At 1109 cm^−1^ in the figure, a new peak can be seen, and the asymmetric stretching vibration of Si-O-Si just corresponds to this peak. This is obvious versus pure PLA and other SAO/PLA composites. All these indicate that the SAO particles have successfully combined with the PLA surface.

### 3.3. Thermal Properties

**Figure 3:**
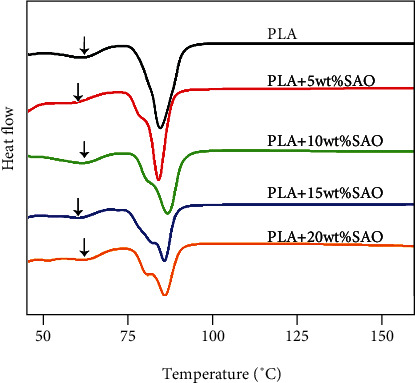
DSC curves of pure PLA, 5 wt% SAO/PLA, 10 wt% SAO/PLA, 15 wt% SAO/PLA, and 20 wt% SAO/PLA.

Nonisothermal curing behaviors of SAO/PLA composites with different amounts of SAO particles were investigated by DSC. DSC traces of SAO/PLA composites containing different amounts of SAO particles are shown in Figure 3. A glass transition occurs near 63°C, and there is a cold crystallization peak near 75°C. The melting peak occurs near 90°C in all samples [17]. The addition of SAO content leads to a melting peak that shifts to the right as a whole, and the melting temperature increased. The glass transition temperature of those samples shows that the blends of SAO and PLA are compatible, and the compatibility of 5 wt% and 15 wt% is better than all other samples.

**Figure 4:**
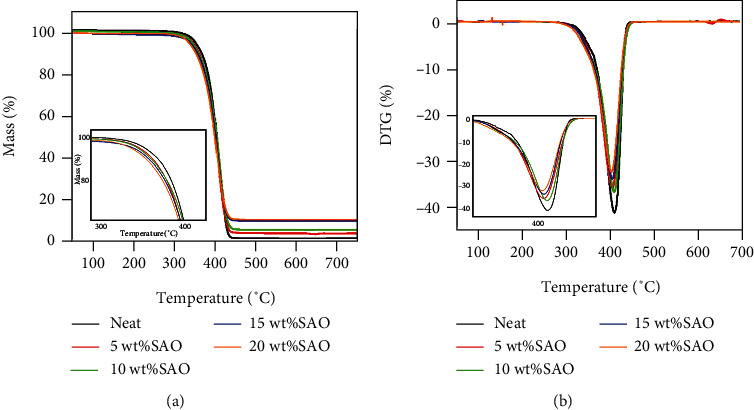
TG (a) and DTG (b) curves of pure PLA, 5 wt% SAO/PLA, 10 wt% SAO/PLA, 15 wt% SAO/PLA, and 20 wt% SAO/PLA.

**Figure 5:**
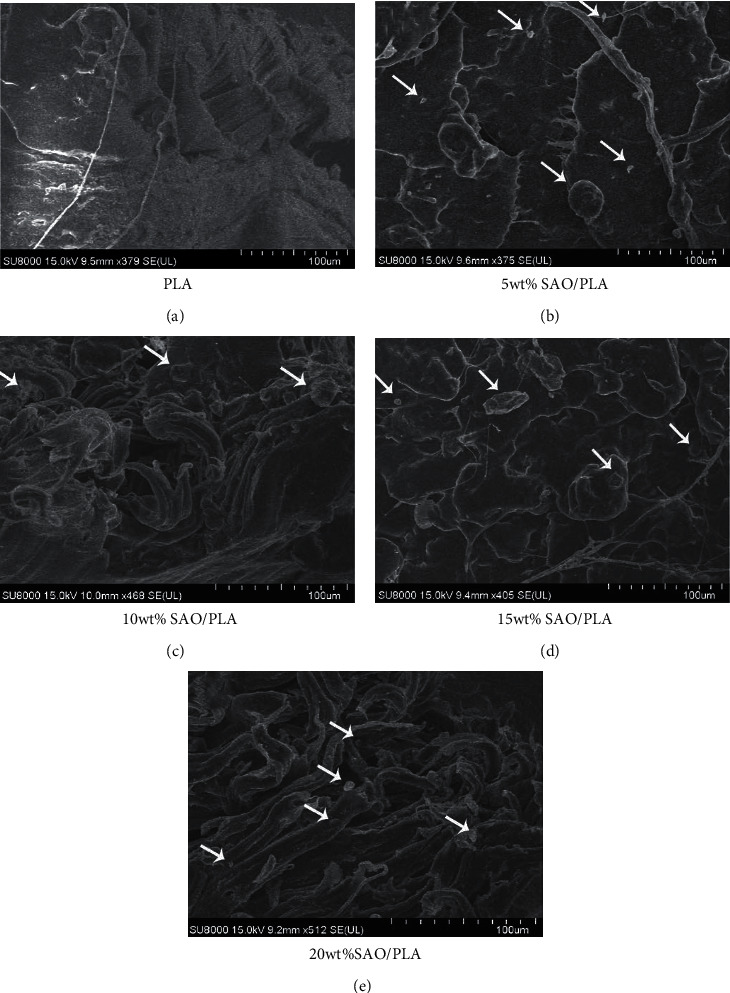
SEM images of fractures for (a) pure PLA, (b) 5 wt% SAO/PLA, (c) 10 wt% SAO/PLA, (d) 15 wt% SAO/PLA, and (e) 20 wt% SAO/PLA.

To investigate the effect of SAO content, we measured the thermal stability of *as*-prepared SAO/PLA composites (Figure 4). *T*_*g*_ first decreases with increasing SAO loadings. The initial weight loss of neat PLA occurred at around 350°C; the lowest temperature is about 320°C. The addition of SAO decreases the thermal stability of PLA, but the rate of decline is slow. This is because hydrogen bonding between SAO and PLA promotes thermal movement of PLA macromolecules by PLA itself. This reduces the energy required to break the chain among the macromolecules [18]. DTG data are plotted in Figure 5(b). The temperature corresponding to the peak temperature of DTG is the maximum weightlessness rate *T*_max_. The *T*_max_ of neat PLA is 423.36°C. The temperature *T*_max_ of the maximum weight loss rate of SAO/PLA composite decreases with addition of SAO because SAO inhibits the crystallization of PLA and decreases the thermal stability. There is a hydroxyl effect from the SAO after treatment with the silane coupling agent.

### 3.4. Mechanical Properties

**Figure 6:**
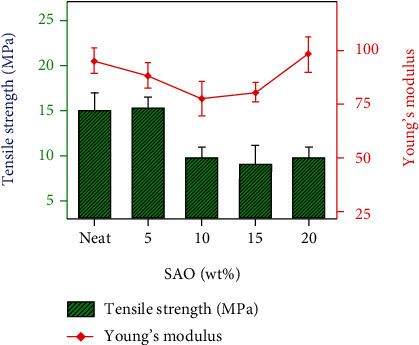
Effect of SAO loading on the tensile properties of PLA.

To investigate the mechanical properties of the *as*-prepared SAO/PLA composites, we measured the tensile strength, fracture elongation, and modulus of elasticity (Table 1, Figure 6). The tensile strength of SAO/PLA composites increases with 5 wt% SAO particles and then decreases at higher contents. This suggests that the tensile strength of the composites is impacted when the crystallinity of the system decreases [19–21]. The fracture elongation decreased when more SAO is added to the composite while the elastic modulus decreases. The toughness and fracture elongation decrease with increasing SAO content. The best overall performance occurs in the SAO/PLA composites containing 5 wt% SAO: 2.7% increase in tensile strength, 29.8% increase in fracture elongation, and 6.3% decrease in fracture elongation. These results indicate that the final mechanical properties of luminescent composite are highly influenced by the addition of SAO [22, 23].

### 3.5. Micromorphology

Tensile fracture SEM images with different amounts of SAO particles are shown in Figure 5. The fracture surface of pure PLA is shown in Figure 5(a), the fracture is relatively flat, smooth, and arranged regularly, with only some shapes like the lines of rivers, and it can be seen from the above situation that it conforms to the characteristics of brittle failure. The SEM image (Figure 5(b)) shows some tiny SAO particles on the surface of pure PLA, which indicates that SAO particles in the composite agglomerate due to its relatively high surface energy. The SEM images of SAO/PLA (Figures 5(c)–5(e)) show a moderately fair dispersion and uniform distribution of PLA at the micro level; some agglomerations on a pure PLA surface can be observed. However, the filaments may be formed during the preparation of the composites via hydrogen bonding, and the orientation of treated SAO particles is obvious under external force [24]. While SAO particles are uniformly distributed into the PLA matrix, some agglomerations of SAO particles are also present, and this may negatively affect the tensile properties. However, the relatively random distribution of SAO particles is favorable and helps the luminous SAO/PLA composite radiate green light after excitation [25].

### 3.6. Fluorescence Properties

**Figure 7:**
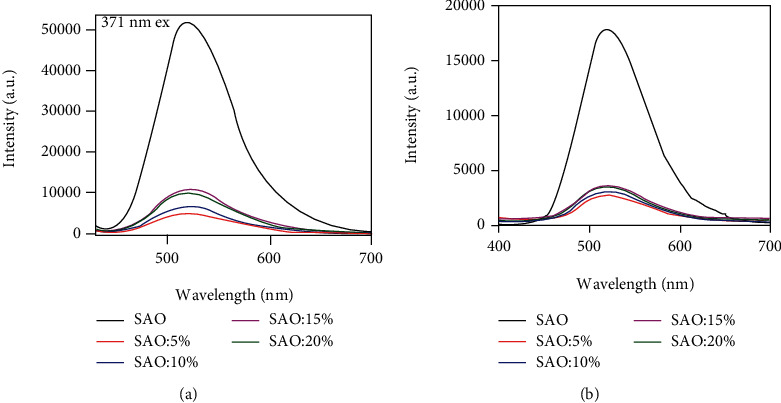
Fluorescence spectra (a) and phosphor spectra (b) of SAO, 5 wt% SAO/PLA, 10 wt% SAO/PLA, 15 wt% SAO/PLA, and 20 wt% SAO/PLA.

The fluorescence spectra and phosphorescence spectra of pure SAO are shown in Figure 7 and include 5 wt% SAO/PLA, 10 wt% SAO/PLA, 15 wt% SAO/PLA, and 20 wt% SAO/PLA composites. The above fully shows that it can be excited by the lowest 320 nm and the highest 400 nm broadband, and the highest excitation intensity is exactly 360 nm in this range. By observing the emission spectra of phosphorescence and fluorescence, it can be found that the SAO particles have a maximum continuous broadband band at 524 nm on both spectra, but it is not difficult to find that the amplitude of fluorescence emission spectrum is significantly higher than that of phosphorescence emission spectrum (Figures 7(a) and 7(b)).  Figure 7(a) shows that the fluorescence emission spectra of the composites are similar to those of SAO particles. This indicates that the composite does not change the crystal phase of SrAl_2_O_4_:Eu^2+^ and Dy^3+^, which belongs to the energy transition between the 4f^6^5d^1^ excited state and the 4f^7^ ground state of Eu^2+^ ions [26]. For the same quality samples, the luminescence intensity increased upon addition of SAO content, but the 15 wt% SAO composites have the best luminescence intensity. This indicates that increasing the SAO content does not further increase the luminous intensity. No special emission peak was found for Dy^3+^, indicating that Dy^3+^ is not the luminescent center, but it does prolong the afterglow time as a trapped energy level [20, 27, 28].

**Figure 8:**
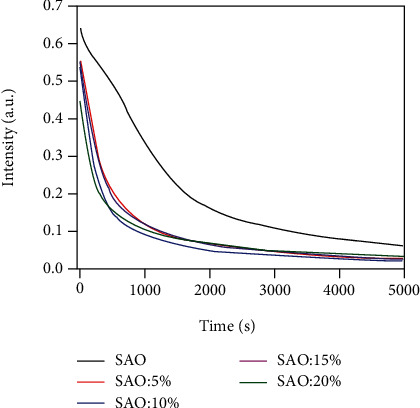
The afterglow decay curve of SAO, 5 wt% SAO/PLA, 10 wt% SAO/PLA, 15 wt% SAO/PLA, and 20 wt% SAO/PLA.

Figure 8 shows the afterglow decay curves of SAO/PLA composites with different amounts of SAO. All samples have the same luminescence decay trend (Figure 8). However, with the gradual increase of the SAO concentration, the initial afterglow intensity will gradually decrease (except of 15 wt%). This is probably because one part of the excitation energy was absorbed and reflected by the PLA crosslinking with the rare-earth-doped particles. However, part of the energy emitted by SrAl_2_O_4_:Eu^2+^ and Dy^3+^ phosphors was also absorbed and reflected by the PLA. Therefore, when the initial PLA concentration is 15 wt %, the cellulose emits more light energy due to the increase of excitation energy. The afterglow decay curve can be divided into two parts, one is fast decay, and the other is slow decay. After the excitation of the low trap energy level ceases, it begins to release electrons by means of thermal disturbance. The low trap level had a shallow depth, low energy, and small binding effects on the electrons [24]. Therefore, it can be known that the reason for the faster decay of the original brightness is related to the faster escape of electrons from the low trap energy level.

**Figure 9:**
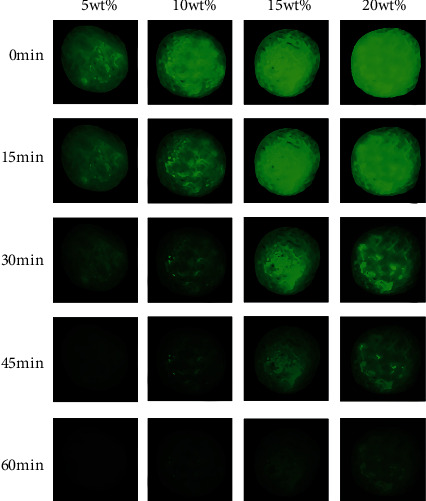
Long afterglow luminescence properties of composites with different SAO contents.

Figure 9 shows the long afterglow luminescence characteristics of composite materials with different SAO contents after sunlight exposure for 2 hours. The luminescence intensity of the composite gradually decreases with time, and it increases with SAO particle content. We concluded that the solvent casting method does not impact the afterglow properties of SrAl_2_O_4_:Eu^2+^ and Dy^3+^.

## 4. Conclusions

The luminous SAO/PLA composites were prepared by a solvent casting method for the first time. The study demonstrated that SAO/PLA composites have the ability to successfully develop materials with fluorescent properties while maintaining good mechanical properties. It only requires SAO particles as a functional agent and PLA as a matrix. All these indicate that the compatibility between SAO and PLA is better improved because of the presence of silane coupling agent. The tenacity, elongation at break, and modulus of the SAO/PLA composites decrease by increasing the SAO content in the composites; 5 wt% SAO composites have the best mechanical properties. The melting and crystallization behavior of the PLA matrix changed upon addition of SAO content. The SAO particles show a moderately fair dispersion and uniform distribution of PLA at the microlevel. The melting and crystallization behavior of the PLA matrix changed after the addition of the SAO particles. Therefore, SAO particles have not been destroyed in the blend with PLA matrix; they retain their luminescent properties. Meanwhile, the decay curves of the composite have a similar behavior to pure particles. By observing the phosphorescence spectra and photoluminescence spectra of the composite SAO particles, we can see that there is a main emission peak at 524 nm of the spectrum, which is consistent with that of pure SAO particles. It can also be seen that the attenuation curve reflected by the composite material is highly similar to that of pure particles. Meanwhile, 5 wt% SAO composite has better fluorescence properties. In conclusion, by controlling the addition of SAO particles, a SAO/PLA composite can be prepared with excellent mechanical properties and useful fluorescence performance. This product may have utility in materials applications. In the future studies, works can be carried out in the following ways: (1) test more different concentration ratios of SAO to find the best one. (2) Better applications should be found for the materials studied in this paper.

## Figures and Tables

**Table 1:** Tensile properties of tensile strength, elongation at break, and modulus of elasticity.

SAO (wt%)	Tensile strength (MPa)	Fracture elongation (%)	Modulus of elasticity (MPa)
0	14.94 ± 4.06	491.95 ± 284.06	94.78 ± 6.43
5	15.31 ± 5.32	699.61 ± 416.29	88.83 ± 6.96
10	9.74 ± 1.36	478.93 ± 143.38	77.44 ± 15.86
15	9.07 ± 2.22	408.08 ± 82.63	80.53 ± 17.76
20	9.82 ± 2.13	262.87 ± 45.79	98.04 ± 7.46

## Data Availability

The datasets used and/or analyzed during the current study are available from the corresponding author on reasonable request.
